# Graft thrombosis after coronary artery bypass surgery and current practice for prevention

**DOI:** 10.3389/fcvm.2023.1125126

**Published:** 2023-03-08

**Authors:** Lamia Harik, Roberto Perezgrovas-Olaria, Giovanni Soletti, Arnaldo Dimagli, Talal Alzghari, Kevin R. An, Gianmarco Cancelli, Mario Gaudino, Sigrid Sandner

**Affiliations:** ^1^ Department of Cardiothoracic Surgery, Weill Cornell Medicine, New York, NY, United States; ^2^ Department of Cardiac Surgery, Medical University of Vienna, Vienna, Austria

**Keywords:** coronary artery bypass grafting, dual antiplatelet therapy (DAPT), aspirin, graft failure, antithrombotics, graft thrombosis

## Abstract

Coronary artery bypass grafting (CABG) is the most frequently performed cardiac surgery worldwide. The reported incidence of graft failure ranges between 10% and 50%, depending upon the type of conduit used. Thrombosis is the predominant mechanism of early graft failure, occurring in both arterial and vein grafts. Significant advances have been made in the field of antithrombotic therapy since the introduction of aspirin, which is regarded as the cornerstone of antithrombotic therapy for prevention of graft thrombosis. Convincing evidence now exists that dual antiplatelet therapy (DAPT), consisting of aspirin and a potent oral P2Y_12_ inhibitor, effectively reduces the incidence of graft failure. However, this is achieved at the expense of an increase in clinically important bleeding, underscoring the importance of balancing thrombotic risk and bleeding risk when considering antithrombotic therapy after CABG. In contrast, anticoagulant therapy has proved ineffective at reducing the occurrence of graft thrombosis, pointing to platelet aggregation as the key driver of graft thrombosis. We provide a comprehensive review of current practice for prevention of graft thrombosis and discuss potential future concepts for antithrombotic therapy including P2Y_12_ inhibitor monotherapy and short-term DAPT.

## Introduction

Coronary artery bypass grafting (CABG) is the most frequently performed cardiac surgery worldwide, and over 300,000 procedures are performed in the United States alone per year (1). The reported long-term incidence of graft failure ranges from 10%–50%, depending upon the type of conduit used, with the highest incidence found in vein grafts (2–5). Antiplatelet therapy is the cornerstone of medical therapy after CABG in order to prevent graft failure, and in particular to prevent early failure, which occurs secondary to graft thrombosis (5, 6). Here, we review the mechanism of graft thrombosis after CABG and provide a comprehensive overview of current antithrombotic strategies for its prevention.

## Grafts for CABG and mechanisms of graft failure

Grafts used for CABG are either arterial grafts that are typically harvested from the chest wall [internal thoracic arteries (ITA)], arms [radial artery (RA)], and abdomen [right gastroepiploic (RGEA)], or vein grafts harvested from the lower extremities (saphenous veins). Despite lower patency rates compared to arterial grafts, vein grafts remain the most frequently used graft in CABG, with a usage rate that approaches 90% globally (7).

Graft failure represents complete occlusion of the graft preventing blood flow to the portion of the heart targeted for revascularization. Morphological and functional characteristics of the graft, as well as the target vessel (including degree of stenosis, vessel diameter, and atherosclerotic burden of the distal vascular bed), technical factors (such as harvesting technique, intraoperative graft storage and preservation, and anastomotic technique), and underlying patient-related atherosclerotic risk contribute to the multifactorial process of graft failure.

**Figure 1:**
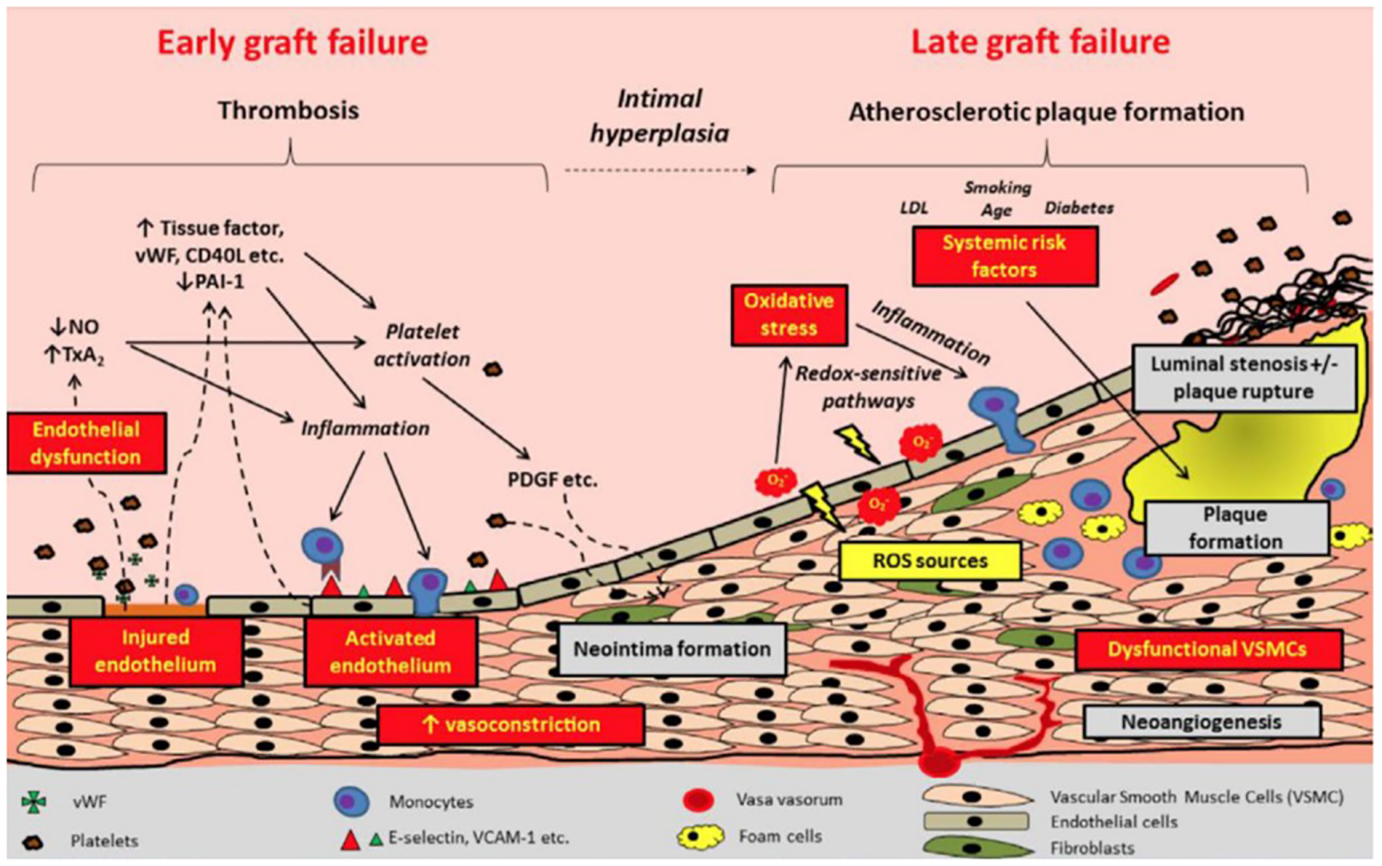
Pathophysiology and timeline of graft failure. Reproduced with permission from Gaudino et al. LDL, low density lipoprotein; NO, nitric oxide; PAI-1, plasminogen activator inhibitor 1; PDGF: ROS, reactive oxygen species; TxA2, thromboxane A2; VCAM, vascular cell adhesion molecular; VSMC, vascular smooth muscle cells; vWF, von Willebrand Factor.

The dominant mechanism of graft failure varies by type of graft and with time from surgery. Early graft failure, or graft occlusion occurring within the first month after CABG, is characterized by acute thrombosis, and its prevention is the target of antithrombotic medications after CABG. Both arterial and vein grafts may fail due to acute thrombosis; however, thrombotic graft occlusion occurs more frequently in the latter. Vein graft failure occurring beyond the first month after CABG is characterized by intimal hyperplasia and accelerated atherosclerosis (Figure 1) (6, 8, 9). Competitive flow through the native coronary artery is a main mechanism for occlusion for arterial grafts (10).

## Mechanism of graft thrombosis

The initial injury underlying the process leading to acute graft thrombosis is that to the vascular endothelium. Disruption of endothelial integrity is typically incurred during graft harvesting due to mechanical trauma, and, although also described in free arterial grafts (11), is predominantly observed in vein grafts (8). Anastomotic imperfections and consequent turbulence in graft flow, size mismatch between the graft and the coronary target vessel, pre-existing graft pathology, and postoperative hypercoagulability and systemic inflammatory reaction may also cause acute graft thrombosis (8, 9).

Endothelial injury leads to activation of a platelet-mediated thrombotic cascade (Figure 1) (5). Pro-inflammatory mediators are released from the damaged endothelium and smooth muscle cells, triggering adhesion and aggregation of leukocytes, platelets, and fibrin to exposed extracellular matrix proteins, thus promoting thrombus formation (8, 12). Activation of the extrinsic coagulation cascade follows (9, 12). Impaired endothelial function results in reduced bioavailability of prostacyclin and nitric oxide, which in turn lead to vasoconstriction and stasis, thereby further promoting platelet adherence and thrombus formation (12–15).

## Antithrombotics in the prevention of graft thrombosis

### Aspirin

**Figure 2:**
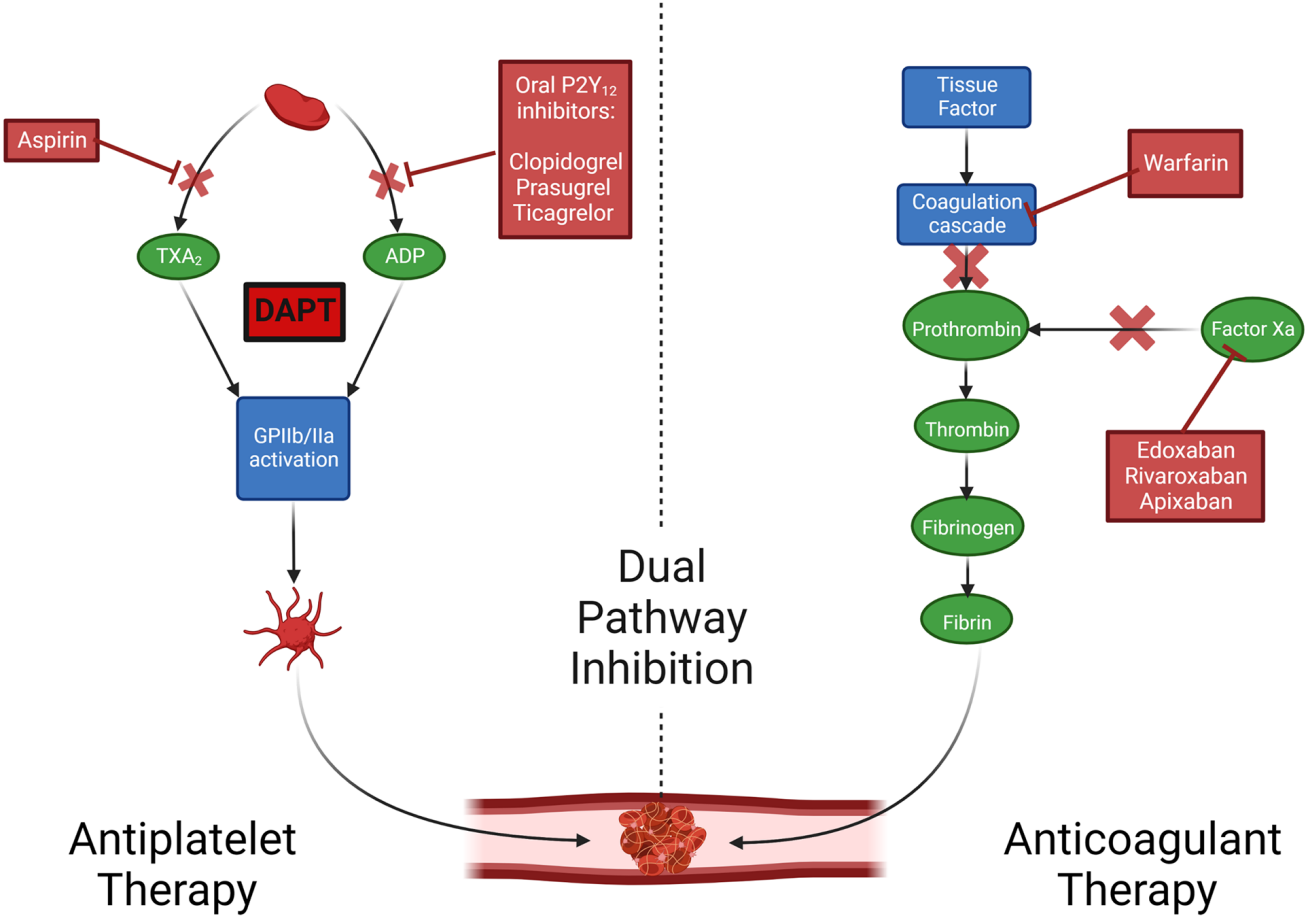
Mechanisms of action of oral antithrombotics. Created with BioRender.com. Adapted with permission from Collet et al. ADP, adenosine diphosphate; DAPT, dual antiplatelet therapy; TxA2, thromboxane A2.

Aspirin is a non-selective, irreversible cyclooxygenase inhibitor that prevents downstream prostaglandin and thromboxane A_2_ synthesis, thus inhibiting platelet aggregation (Figure 2). It is currently the antithrombotic agent of choice after CABG; however, the data that supports the use of aspirin monotherapy after CABG to prevent graft thrombosis is decades old. Lorenz et al. in a 1984 randomized clinical trial (RCT) comparing 100 mg aspirin vs. placebo administered 24 h after CABG in 60 patients demonstrated increased vein graft patency in the aspirin group at four-month angiographic follow-up (90% vs. 68%; *P* = 0.012) (16). In the largest placebo-controlled aspirin trial, Goldman et al. randomized 772 CABG patients to three different aspirin strategies (aspirin once daily; aspirin three times daily; aspirin plus dipyridamole three times daily), with one aspirin dose given 12 h before CABG, and the assigned regimen initiated six hours after CABG and continued for one year (17). All aspirin regimens significantly improved angiographic graft patency early (within 60 days of CABG) compared with placebo (93.5%, 92.3%, 91.9% vs. 85.2%; *P* < 0.05) (17). A 1993 meta-analysis of seventeen RCTs (1,443 patients) showed that aspirin significantly reduced graft occlusion compared with placebo (odds ratio [OR] 0.60, 95% confidence interval [CI] 0.51–0.71; *P* < 0.0001), with the best time for aspirin initiation being within six hours of surgery (18). A meta-analysis (19) of five RCTs comparing 50–100 mg and 300–325 mg dosing of aspirin found no significant difference in the association of aspirin dose with graft patency, although a non-significant trend toward improved graft patency with 300–325 mg was observed [relative risk (RR) 0.74, 95% CI 0.52–1.06; *P* = 0.10]. Two small RCTs evaluating the effect of more frequent administration of low-dose aspirin (81–100 mg) vs. once-daily administration of high-dose aspirin (200–325 mg) showed that more frequent dosing was more effective in suppressing serum TXB2 formation (20) and prevented platelet activation associated with enhanced platelet turnover (21), which has been postulated as a likely etiology for reduced efficacy of low-dose aspirin (“aspirin resistance”) in the early postoperative phase after on-pump CABG (22). However, whether more frequent aspirin dosing reduces early graft thrombosis has not yet been tested in an RCT.

There are currently discrepant recommendations for timing and dosing of aspirin after CABG. The 2021 American College of Cardiology (ACC)/American Heart Association (AHA)/and Society for Cardiovascular Angiography and Interventions (SCAI) Guideline for Coronary Artery Revascularization recommends 100–325 mg aspirin daily initiated within six hours postoperatively and then continued indefinitely to reduce the occurrence of vein graft closure and adverse cardiovascular events (class of recommendation [COR] I, level of evidence [LOE] A) (23). The 2018 European Society of Cardiology (ESC)/European Association for Cardio-Thoracic Surgery (EACTS) guidelines on myocardial revascularization (24) recommend starting 75–100 mg aspirin within 24 h of surgery or as soon as there is no concern for bleeding (COR I, LOE C).

### Dual antiplatelet therapy

P2Y_12_ receptor inhibitors inhibit binding of adenosine diphosphate (ADP) to its platelet P2Y_12_ receptor, preventing the ADP-mediated activation of the GPIIb/IIIa complex and subsequent platelet aggregation (25). Adding an oral P2Y_12_ inhibitor to aspirin [dual antiplatelet therapy (DAPT)] leads to enhanced platelet inhibitory effects (26). The evolution of DAPT in the setting of increased thrombotic risk (after acute coronary syndrome [ACS] or percutaneous coronary intervention [PCI]), indicating that intensified platelet inhibition reduces ischemic events and mortality, has led to interest in pursuing this strategy for prevention of graft thrombosis after CABG.

An overview of oral P2Y_12_ inhibitors is presented in Table 1. Clopidogrel is an irreversible P2Y_12_ inhibitor and shows variable interindividual response with about one-third of patients having inadequate platelet inhibitory effects. Importantly, such patients who show high platelet reactivity with use of clopidogrel have an increased risk of thrombotic events (27). Clopidogrel response variability is attributed to multiple factors, including genetic (i.e., loss of function alleles for the CYP2C19 enzyme), drug-drug interactions, and patient comorbidities (such as chronic kidney disease, and diabetes) (28, 29). Ticagrelor is a reversible P2Y_12_ inhibitor with a rapid onset and offset of action (30) and has shown increased platelet inhibition compared with clopidogrel. In patients with ACS and those undergoing PCI, platelet inhibition with ticagrelor DAPT is associated with a greater reduction in ischemic events compared with clopidogrel DAPT; however, this is achieved at the cost of an increased risk of bleeding (26, 31, 32). Prasugrel is a thienopyridine prodrug that acts as an irreversible P2Y_12_ inhibitor. Its hepatic metabolic conversion requires only one oxidation step (30), and so it shows less variability in interindividual response than clopidogrel, as well as greater inhibition of platelet activation (33).

**Table 1:** Characteristics of oral P2Y_12_ inhibitors.

Drug Characteristics	Ticagrelor	Clopidogrel	Prasugrel
Chemical group	Cyclopentyl-Triazolopyrimidine	Thienopyridine	Thienopyridine
Prodrug	No	Yes	Yes
Conversion to active drug	N/A	Two-step (CYP450, CYP2C19)	One-step (CYP450)
Metabolism	Hepatic	Hepatic	Hepatic
Platelet inhibition type	Reversible	Irreversible	Irreversible
Inhibition of platelet activation	80%–90%	50%–70%	80%–90%
Time to platelet inhibition	30 min	2–4 h	1 h
Time to platelet function recovery	24–48 h	5 days	7 days
Transfuse to counteract	No	Yes	Yes
Reversal agent	PB2452*^1^	None	None
Maintenance dose after CABG	90 mg bid	75 mg qd	10 mg qd
Recommended aspirin dose for DAPT	75–100 mg qd	No recommendation	Norecommendation
Randomized evidence for prevention of graft thrombosis	Yes	Yes	No

bid, twice a day; CABG, coronary artery bypass grafting; DAPT, dual anti-platelet therapy; mg, milligrams; N/A, not applicable; qd, daily.

* Bentracimab, not yet in use, phase 2B.

^1^ Bhatt DL, Pollack CV, Weitz JI, et al. Antibody-Based Ticagrelor Reversal Agent in Healthy Volunteers. N Engl J Med. 2019;380(19):1825–1833. doi:10.1056/NEJMoa1901778.

**Figure 3:**
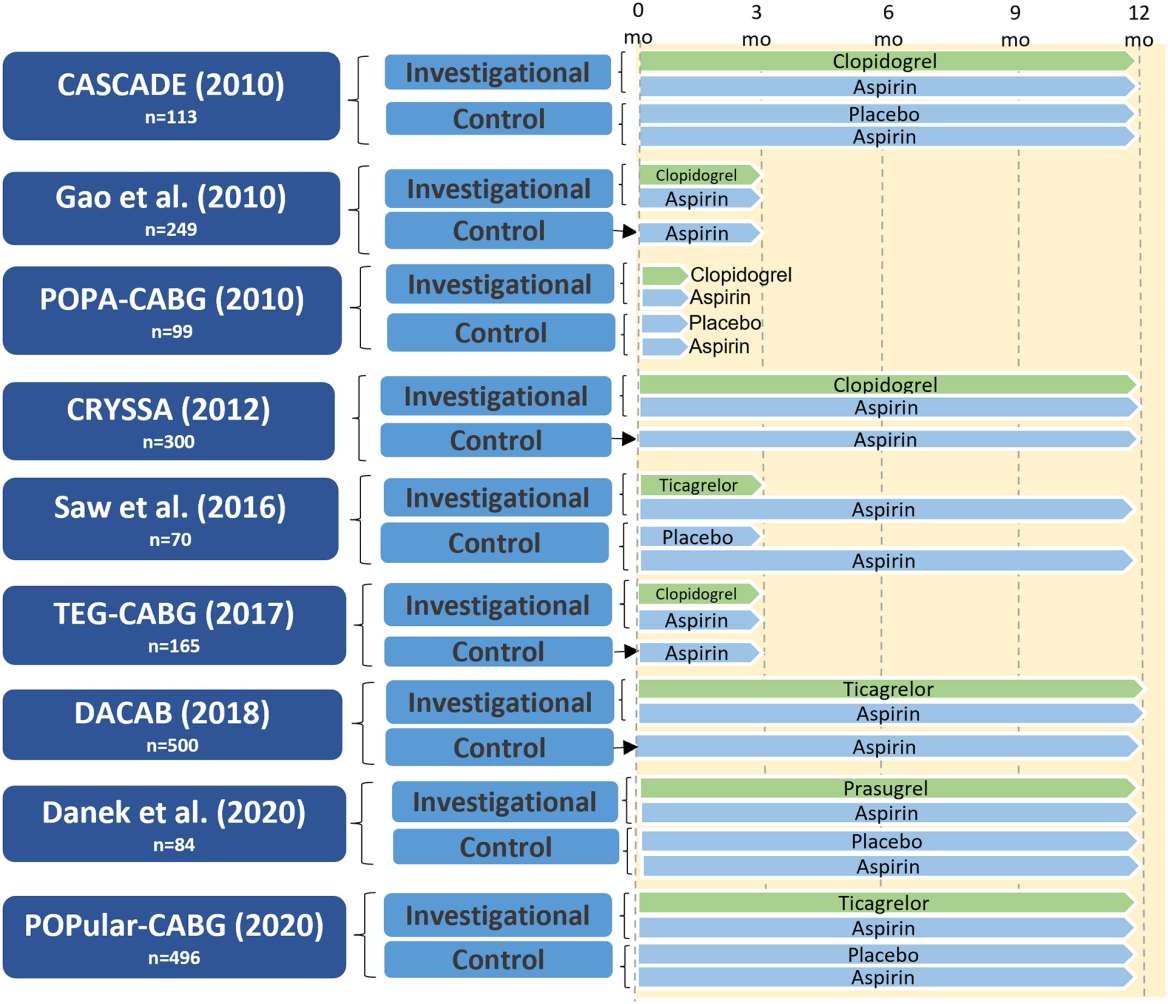
Study design of randomized controlled trials of dual antiplatelet therapy versus aspirin in patients undergoing CABG with protocol-defined graft imaging. CABG, coronary artery bypass grafting; CASCADE, clopidogrel after surgery for coronary artery disease; CRYSSA, prevention of coronary artery bypass occlusion after off-pump procedures; DACAB, dual ticagrelor plus aspirin antiplatelet strategy after coronary artery bypass grafting; DAPT, dual antiplatelet therapy; mo, months; POPA-CABG, preoperative aspirin and postoperative antiplatelets in coronary artery bypass grafting; POPular CABG, effect of adding ticagrelor to standard aspirin on saphenous vein graft patency in patients undergoing coronary artery bypass grafting; TEG-CABG, thrombelastographic hypercoagulability and antiplatelet therapy after coronary artery bypass surgery.

The study design of CABG RCTs of DAPT vs. aspirin that included protocol-defined graft imaging are presented in Figure 3. The placebo-controlled CASCADE trial (34) included 113 patients and compared aspirin 81 mg twice daily with aspirin 81 mg twice daily plus clopidogrel. The majority of patients (96%) underwent on-pump CABG. Compared with aspirin, clopidogrel DAPT did not significantly reduce overall graft patency (95.5% vs. 95.2%; *P* = 0.90) or vein graft patency (93.2% vs. 94.3%; *P* = 0.69) one year after CABG (34). In the CRYSSA trial that included 300 off-pump CABG patients, clopidogrel DAPT was associated with a reduced one-year vein graft occlusion rate compared with aspirin 100 mg (7.4% vs. 13.1%; *P* = 0.04) (35). In a 2013 meta-analysis of eleven studies (five RCTs, six observational) comparing clopidogrel DAPT with aspirin (25,728 patients), clopidogrel DAPT was associated with significantly reduced vein graft occlusion rates (RR 0.59, 95% CI 0.43–0.82; *P* = 0.02), and with an increased risk of major bleeding events (RR 1.17, 95% CI 1.00–1.37; *P* = 0.05) (36). A subgroup analysis in studies using off-pump CABG found that clopidogrel DAPT was associated with a reduced 14% reduced risk of vein graft occlusion compared to aspirin alone (two studies, 560 patients). Based on these data the 2015 AHA scientific statement on secondary prevention after coronary artery bypass graft surgery (37) recommend DAPT with aspirin (81–162 mg daily) and clopidogrel after off-pump CABG to reduce graft occlusion (COR I, LOE A). A meta-analysis by Nocerino et al. of five RCTs (38) comparing clopidogrel DAPT with aspirin alone (958 patients) also found an association between aspirin and increased vein graft occlusion (OR 1.70, 95% CI 1.20–2.40). Notably, this study did not find any effect of clopidogrel DAPT on arterial graft occlusion (OR 1.17, 95% CI 0.54–2.56).

The two larger RCTs investigating the effect of ticagrelor (90 mg twice daily) plus aspirin (80–100 mg once daily) with aspirin alone have yielded conflicting results (39, 40). The three-arm DACAB (39) trial compared one-year ticagrelor DAPT vs. single antiplatelet therapy (aspirin or ticagrelor) in 500 CABG patients. At one year, the ticagrelor DAPT group had a significantly lower incidence of vein graft failure compared with aspirin alone (11.3% vs. 23.5%; RR 0.48, 95% CI 0.31–0.74; *P* < 0.001). The trial was performed in an exclusively Chinese population, and 75.8% of patients underwent off-pump CABG, thus limiting generalizability of the findings. In the POPular-CABG trial (40) that included 496 patients the one-year rate of vein graft occlusion was similar between the two trial arms (ticagrelor DAPT: 9.6% vs. aspirin: 10.1%; OR 0.87, 95% CI 0.49–1.55; *P* = 0.64). However, the trial was limited by poor compliance with the allocated treatment, as 37.8% of patients in the ticagrelor arm permanently discontinued the study medication during the 12 months of treatment.

A meta-analysis of 22 studies and 20,315 patients by Cardoso et al. (41) comparing DAPT to aspirin (clopidogrel DAPT: 20 studies; ticagrelor DAPT: two studies) found that vein graft occlusion was significantly lower with DAPT (nine RCTs, OR 0.64, 95% CI 0.50–0.83; *P* < 0.01), but major bleeding events were significantly increased (eight RCTs, OR 1.31; 95% CI 1.02–1.68; *P* = 0.03). A network meta-analysis (42) of 20 RCTs (4,803 patients) and nine different antithrombotic strategies found that the use of either ticagrelor DAPT [two RCTS, OR 0.50, 95% CI 0.31–0.79; number needed to treat (NNT) = 10] or clopidogrel DAPT (seven RCTs, OR 0.60, 95% CI 0.42–0.86, NNT = 19) reduced vein graft failure compared with aspirin alone. However, in all study-level meta-analyses, there was considerable heterogeneity in drug dosing, duration of treatment and follow-up, as well definitions of vein graft failure used. Most recently, an individual patient data meta-analysis of four RCTs (1,316 patients) demonstrated that ticagrelor DAPT was associated with a significantly lower incidence of vein graft failure compared with aspirin alone (11% vs. 20%; OR 0.51, 95% CI 0.35–0.74; *P* < 0.001) and this finding was consistent across all prespecified subgroups, including those undergoing off-pump CABG (43). Ticagrelor DAPT was associated with a significantly lower incidence of any graft failure compared with aspirin (7.5% vs. 13.6%; OR 0.52, 95% CI 0.38–0.72; *P* < 0.001), and the finding was consistent when stratified by arterial (OR 0.52, 95% CI 0.27–1.04) vs. vein grafts (OR 0.51, 95% CI 0.35–0.74) (p_int _= 0.93). Notably, the median treatment duration with ticagrelor DAPT was one year, and ticagrelor DAPT was associated with an increased risk of clinically important bleeding events compared with aspirin alone (8.7% vs. 13.3%; OR 2.98, 95% CI 1.99–4.47; *P* < 0.001).

In the only placebo-controlled RCT testing a DAPT strategy including prasugrel vs. aspirin Danek et al. reported no difference in the incidence of optical coherence tomography-detected vein graft thrombus in 84 patients one year after CABG (15).

The 2021 ACC/AHA/SCAI guidelines give a COR IIb, LOE B-R (23) recommendation for the use of DAPT with aspirin and ticagrelor or clopidogrel for one year in selected patients to improve vein graft patency compared with aspirin alone; no such recommendation currently exists in European guidelines. However, recommendations are given in European guidelines for use of DAPT after CABG in patients with ACS and those who have recently received coronary stents (24, 44, 45), and selected patients with stable coronary disease undergoing off-pump CABG or endarterectomy (44).

### P2Y_12_ inhibitor monotherapy

**Figure 4:**
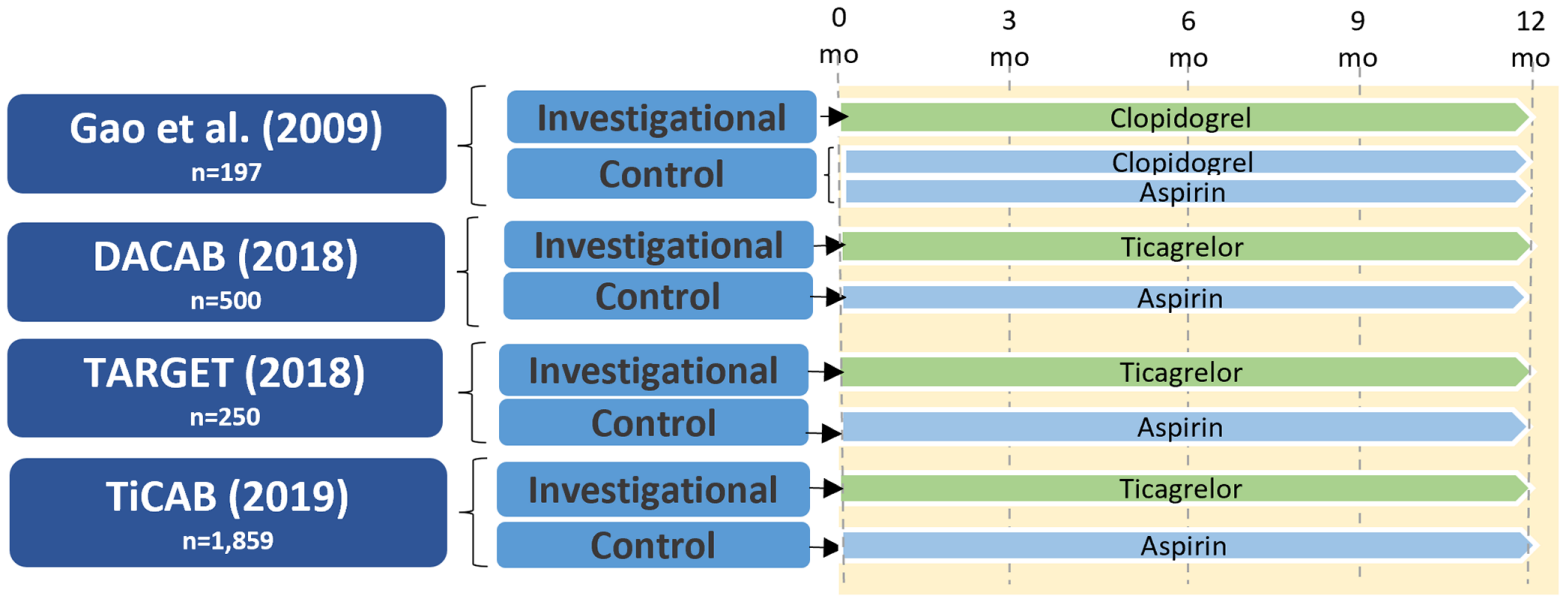
Study design of randomized controlled trials of P2Y_12_ inhibitor monotherapy versus aspirin in patients undergoing CABG. CABG, coronary artery bypass grafting; DACAB, dual ticagrelor plus aspirin antiplatelet strategy after coronary artery bypass grafting; mo, months; TARGET, ticagrelor antiplatelet therapy to reduce graft events and thrombosis; TiCAB, randomized trial of ticagrelor vs. aspirin in patients after coronary artery bypass grafting.

A small number of RCTs examined the role of P2Y_12_ inhibitor monotherapy after CABG, and are summarized in Figure 4. Gao et al. randomized 197 patients to clopidogrel DAPT vs. clopidogrel monotherapy and found no significant differences between the groups in left ITA and vein graft patency at either one month (left ITA: 99.0% vs. 98.9%; *P* = 0.77; vein graft: 98.1% vs. 98.2%; *P* = 0.73) or one year after CABG (left ITA: 96.9% vs. 97.8%; *P* = 0.91; vein graft: 93.5% vs. 96.3%; *P* = 0.25) (46).

In the DACAB trial (39) ticagrelor monotherapy did not significantly increase vein graft patency compared with aspirin one year after CABG (82.8% vs. 76.5%; RR 0.73, 95% CI 0.51–1.06; *P* = 0.10). The TARGET trial randomizing 250 patients after CABG to ticagrelor monotherapy (90 mg twice daily) or aspirin (81 mg twice daily) similarly found no difference between groups in the incidence of vein graft occlusion at one year (17.4% vs. 13.2%, aspirin vs. ticagrelor; *P* = 0.30) (47). The TiCAB trial randomized 1,859 CABG patients to either ticagrelor monotherapy or aspirin for one year and reported no difference between groups in the incidence of the primary composite efficacy endpoint of cardiovascular death, MI, stroke, or repeat revascularization (ticagrelor: 9.7%, aspirin: 8.2%; HR 1.10, 95% CI 0.87–1.62; *P* = 0.28) or the secondary safety endpoint of major bleeding (ticagrelor: 3.7%, aspirin: 3.2%; HR 1.17, 95% CI 0.71–1.92; *P* = 0.53); however, this study did not include graft failure as an outcome (48).

### Anticoagulant therapy and dual pathway inhibition

While phenprocoumon improved graft patency vs. placebo in a 1981 RCT of 89 patients eight weeks after CABG (49), subsequent RCTs did not demonstrate a benefit of vitamin K antagonists over antiplatelet therapy in preventing graft occlusion (50, 51). In the sub-study of the COMPASS trial (52) that included 1,448 patients undergoing CABG, the factor Xa inhibitor rivaroxaban alone or in combination with aspirin did not reduce the one-year incidence of graft failure compared with aspirin alone (rivaroxaban plus aspirin vs. aspirin: 9.1% vs. 8.0%; OR: 1.13, 95% CI 0.82–1.57; *P* = 0.45; rivaroxaban alone vs. aspirin: 7.8% vs. 8.0%; OR: 0.95, 95% CI: 0.67–1.33; *P* = 0.75). This points to graft thrombosis as a primarily platelet-driven event, and antithrombotic strategies including oral anticoagulants are currently not recommended for prevention of graft failure.

## Graft failure and clinical events

The association between graft failure and clinical events is complex, and may vary by type of graft and the area of subtended myocardium supplied by the failed graft (10). Studies reporting on the association of vein graft occlusion with clinical events have shown discrepant results (10). Early prospective series with per-protocol angiography reported an association of vein graft occlusion with recurrence of angina (53, 54) and mortality (4, 53). In the PREVENT IV trial vein graft failure was associated with repeat revascularization, but not with death or myocardial infarction (55). In the RAPS trial, the risk of death, myocardial infarction or repeat revascularization was significantly higher in patients with vein graft failure (56). Overall, the majority of studies reporting an association between graft status and clinical events have shown an association of graft failure with non-fatal cardiac events rather than death. The discrepancy in findings is likely due to differences in study size, use of clinically driven vs. per-protocol imaging, follow-up, and different definitions of graft failure. Further investigation is needed to elucidate the association between graft occlusion and clinical events.

## Gaps in knowledge and future directions

Women and racial minorities are underrepresented in cardiovascular RCTs (57–59), and a key limitation of current recommendations for antithrombotic therapy after CABG therefore is their reliance on data derived from prevalently white male populations (60, 61). Platelet count, morphology, activation and aggregation have been shown to differ by sex, age, and ethnicity/race (62–64). In addition, sex-specific differences in pharmacokinetics and pharmacodynamics of antithrombotic drugs may exist (65). Women have increased bleeding times, higher baseline platelet reactivity, and more potent ADP-induced platelet aggregation than men (65, 66). Ticagrelor exposure has been shown to be higher in women, and its half-life is longer (33). Insight into sex-related differences in the efficacy and safety of antithrombotics is essential, as women frequently present with more advanced CAD and have worse outcomes after CABG than men (67, 68).

With an ageing population and rising life expectancy, an increasing proportion of patients undergoing CABG are elderly adults. A higher prevalence of comorbidities and age-related changes in drug metabolism place elderly patients at a higher risk of ischemic events as well as bleeding events (69). The presence of moderate to severe chronic kidney disease (CKD) has been an exclusion criterion in many RCTs. Antiplatelet therapy is challenging in CKD patients as reduced ischemic risk with more potent platelet inhibition is achieved at the expense of increased bleeding risk (70, 71).

Although ticagrelor monotherapy was not associated with a significant difference in the incidence of vein graft failure compared with aspirin in a recent meta-analysis, the direction of the treatment effect pointed to a potential benefit of ticagrelor monotherapy (43). As ticagrelor monotherapy did not increase the risk of bleeding compared with aspirin, P2Y_12_ inhibitor monotherapy may represent a potential alternative for intensified platelet inhibition without added bleeding risk. In the setting of PCI, shortening the duration of DAPT has become the focus of many studies to reduce bleeding risk while preserving ischemic efficacy. Considering that thrombosis is the predominant mechanism of early graft occlusion and typically occurs during the first month after surgery, DAPT in the setting of CABG may prove most beneficial when given short-term, followed by aspirin alone (or a P2Y_12_ inhibitor alone) after a certain period to mitigate the long-term bleeding risk associated with DAPT. An antithrombotic strategy of short-term DAPT after CABG will necessitate evaluation in adequately powered RCTs.
